# Targeting metabolic reprogramming in glioblastoma as a new strategy to overcome therapy resistance

**DOI:** 10.3389/fcell.2025.1535073

**Published:** 2025-02-26

**Authors:** Simona D’Aprile, Simona Denaro, Anna Gervasi, Nunzio Vicario, Rosalba Parenti

**Affiliations:** Section of Physiology, Department of Biomedical and Biotechnological Sciences, University of Catania, Catania, Italy

**Keywords:** metabolism, Warburg effect, lipids, nucleotides, iron, tumor microenvironment, chemotherapy, radiotherapy

## Abstract

Glioblastoma (GBM) is one of the deadliest tumors due to its high aggressiveness and resistance to standard therapies, resulting in a dismal prognosis. This lethal tumor carries out metabolic reprogramming in order to modulate specific pathways, providing metabolites that promote GBM cells proliferation and limit the efficacy of standard treatments. Indeed, GBM remodels glucose metabolism and undergoes Warburg effect, fuelling glycolysis even when oxygen is available. Moreover, recent evidence revealed a rewiring in nucleotide, lipid and iron metabolism, resulting not only in an increased tumor growth, but also in radio- and chemo-resistance. Thus, while on the one hand metabolic reprogramming is an advantage for GBM, on the other hand it may represent an exploitable target to hamper GBM progression. Lately, a number of studies focused on drugs targeting metabolism to uncover their effects on tumor proliferation and therapy resistance, demonstrating that some of these are effective, in combination with conventional treatments, sensitizing GBM to radiotherapy and chemotherapy. However, GBM heterogeneity could lead to a plethora of metabolic alterations among subtypes, hence a metabolic treatment might be effective for proneural tumors but not for mesenchymal ones, which are more aggressive and resistant to conventional approaches. This review explores key mechanisms of GBM metabolic reprogramming and their involvement in therapy resistance, highlighting how metabolism acts as a double-edged sword for GBM, taking into account metabolic pathways that seem to offer promising treatment options for GBM.

## 1 Introduction

Glioblastoma (GBM) is the most common primary brain tumor, exhibiting highly mortality rate due to its aggressiveness and invasiveness (Wirsching et al., 2016). GBM shows an incidence of 3,19/100.000 people per year, with a median overall survival of 15–18 months and, as a result of recurrences, less than 5% of patients survive over 5 years (Ostrom et al., 2019; Alzial et al., 2022). The current therapeutic approach includes surgical resection, followed by radiotherapy and chemotherapy with temozolomide (TMZ) (Wen et al., 2020). However, the hypoxic microenvironment, the presence of blood brain barrier (BBB) and the GBM heterogeneity limit the efficacy of standard therapies. Indeed, besides inter-patient variability, GBM is also characterized by an intra-tumoral heterogeneity, that makes the tumor difficult to treat (Sottoriva et al., 2013; Cammarata et al., 2023). Nowadays, based on gene expression levels, GBM is classified into 3 subtypes: proneural, mesenchymal and classical (Verhaak et al., 2010; Azam et al., 2020). Mesenchymal tumors are more aggressive and show an increased resistance to treatments as compared to proneural subtypes (Bhat et al., 2013). Moreover, during GBM progression, the tumor profile could change from one subtype to another, generally with a proneural-to-mesenchymal transition (PMT), leading to therapy resistance and a poor patient prognosis (Segerman et al., 2016). Thus, it is crucial to investigate the different features and mechanisms for each subtype, to identify new specific therapeutic targets.

Notably, GBM rewires its metabolism increasing the rate of proliferation and invasion (Torrisi et al., 2023), and influencing chemotherapy and radiotherapy resistance, overcoming their antitumoral effects (Zhou and Wahl, 2019; Torrisi et al., 2020). The metabolic reorganization also provides energy and nutrient in a hostile microenvironment, promoting the invasion of healthy tissues and promoting the production of specific metabolites influencing tumor growth (Torrisi et al., 2022). Moreover, metabolism is also reflecting GBM heterogeneity, since different GBM subtypes are characterized by metabolic signatures, stemness and tumor localization (Tardito et al., 2015; Alzial et al., 2022). The most studied GBM metabolic alteration is the Warburg effect. Brain tumor cells prefer glycolysis as compared to oxidative phosphorylation (OXPHOS), even under oxygen-rich conditions (Potter et al., 2016). GBM cells rely on aerobic glycolysis to produce ATP and show an elevated glucose consumption, rapidly metabolized into lactate (Longhitano et al., 2022). This accelerated glycolytic metabolism is supported by enhanced crosstalk between GBM cells and tumor microenvironment (TME), also through monocarboxylate transporters (MCTs) (Miranda-Goncalves et al., 2017). Resident TME cells, such as neurons, neuroglia, immune cells and endothelial cells, are involved in brain metabolic homeostasis and usually enveloped in a hypoxic milieu, thus implementing GBM metabolic adaptability (Dapash et al., 2021; Torrisi et al., 2021; Longhitano et al., 2023).

Beyond an increased glycolysis, many studies revealed that GBM remodels lipid and nucleotide metabolism, acting on different pathways (Pavlova and Thompson, 2016). Recently, it has been demonstrated that metabolic reprogramming contributes to immunosuppression, that is a typical GBM marker (Hernandez et al., 2021). As a matter of fact, lactate induces the immunosuppression, polarizing tumor-associated macrophages (TAMs) towards a pro-tumoral M2 phenotype (Colegio et al., 2014). Thus, the interaction between metabolism and TME cells could be another potential target for GBM therapy.

This review aims at exploring the common metabolic alterations characterizing GBM and highlighting how metabolic reprogramming may influence tumor aggressiveness and proliferation. The typical metabolic reshaping, on the one hand represents a driver for GBM aggressiveness and, on the other hand, is a factor that may drive therapeutic choices. This review highlighted the importance of considering the metabolic features of GBM subtypes to achieve a personalized and effective therapy for cancer patients.

## 2 Metabolic reprogramming in glioblastoma: an advantage in tumor progression

### 2.1 Glucose metabolism

It is well established that the proliferation and aggressiveness of tumor cells are sustained by the prevalence of aerobic glycolysis, also known as the Warburg effect, over OXPHOS (Cortes Ballen et al., 2024; Zhao et al., 2024). In agreement with this aspect, several independent studies on GBM patients highlighted a significant correlation between upregulation of glycolytic genes, low survival and poor prognosis (Alfardus et al., 2017; Stanke et al., 2021; Cortes Ballen et al., 2024; Zhao et al., 2024).

**FIGURE 1:**
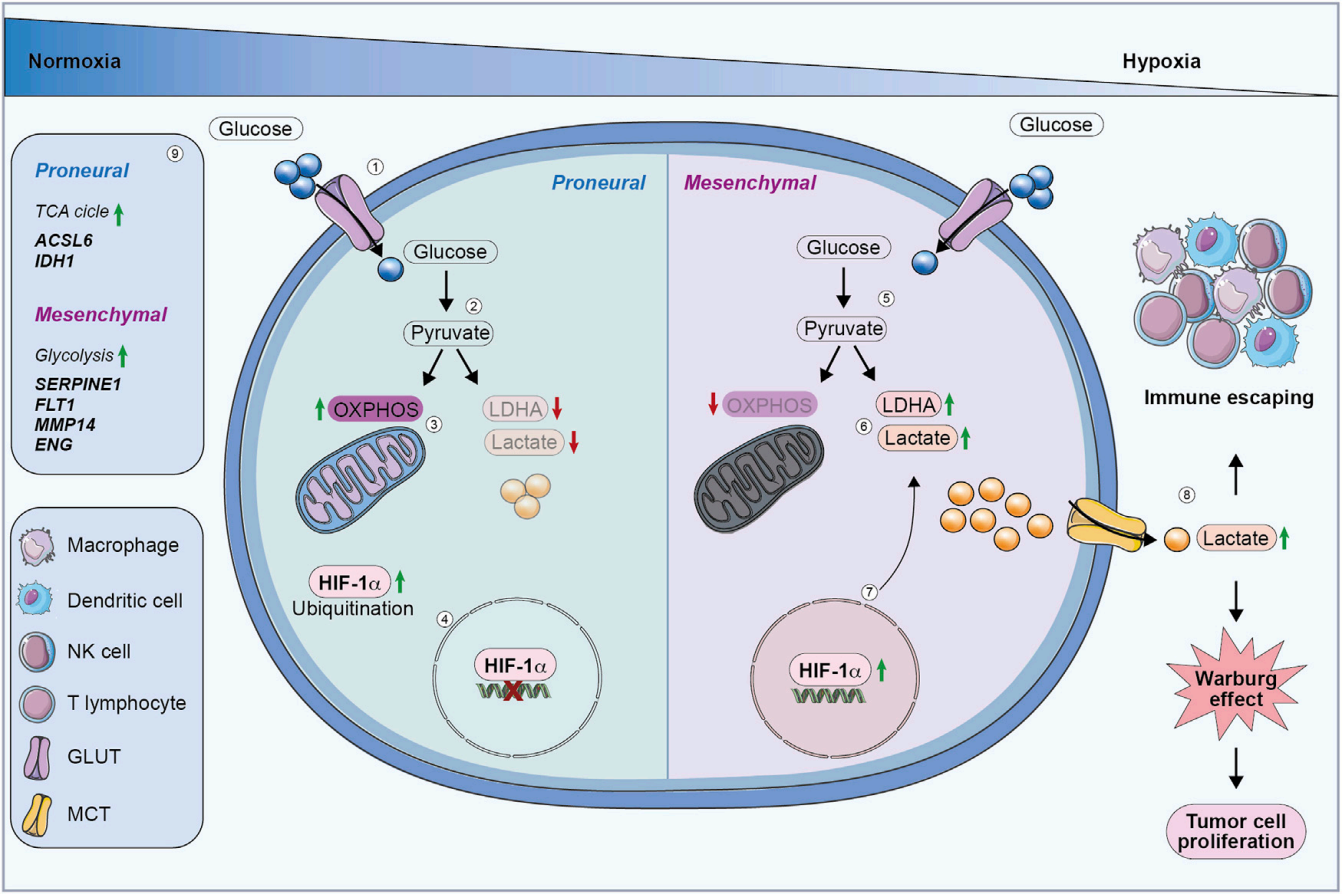
Glucose metabolic reprogramming in GBM cells. (1) In normoxia, proneural cells internalize glucose via glucose transporters. (2) Glucose is metabolized into pyruvate. (3) Proneural cells preferentially use OXPHOS, resulting in reduced lactate production and LDHA downregulation. (4) HIF-1α is degraded, preventing its activity in regulating HREs. (5) Under hypoxia, mesenchymal cells upregulate glycolysis, by converting pyruvate into lactate. (6) Mesenchymal cells produce more lactate, which is accumulated in extracellular environment contributing to the Warburg effect. (7) Hypoxia stabilizes HIF-1α, leading to the activation of genes involved in glycolysis and tumor progression. (8) Increased lactate secretion promotes immune evasion and drives tumor cell proliferation through the Warburg effect. (9) Proneural subtype upregulates genes involved in the TCA cycle, while the mesenchymal subtype enhances glycolysis-related genes. OXPHOS, oxidative phosphorylation; LDHA, lactate dehydrogenase; HIF-1α, hypoxia-inducible factor 1 subunit alpha; HREs, hypoxia-responsive elements; TCA, Tricarboxylic acid cycle.

Hypoxia is a significant activator of the metabolic reprogramming and the Warburg effect. GBM adapts to a poor oxygenated environment by promoting the expression of different transcription factors and epigenetic modifications to survive in these hostile conditions (Miranda-Galvis and Teng, 2020). Particularly, low oxygen levels stimulate the translocation of hypoxia-inducible factor 1 subunit alpha (HIF-1α) from the cytoplasm to the nucleus, where it is stabilized. HIF-1α activation promotes hypoxia response elements (HREs) transcription, and the expression of genes involved in glycolysis, including glucose transporter 1 (GLUT1) and GLUT3, favouring glucose uptake and lactate production (Trejo-Solis et al., 2024). Pyruvate is converted into lactate by lactate dehydrogenase (LDH) through the transfer of a hydride ion from NADH to the carbon C2 of pyruvate. This reaction is regulated by HIF-1α gene that, activating LDH transcription, increases the production and accumulation of lactate in the TME, thus promoting cell proliferation (Figure 1) (Valvona et al., 2016; Domenech et al., 2021).

Consequently, lactate supports the acidic pH of TME, stimulating immune cells recruitment but suppressing the anti-cancer immunity of innate and adaptive immune cells, facilitating tumor invasion (Wang et al., 2020). Lactate inhibits the function and proliferation of cytotoxic T cells, natural killer (NK) cells and dendritic cells, reshaping the polarization of macrophages toward a tumor-supporting M2 phenotype and increasing regulatory T cell (Treg) activity, further enhancing immune evasion (Figure 1) (Calcinotto et al., 2012; El-Kenawi et al., 2019). Mesenchymal GBM secretes higher levels of lactate as compared to other subtypes, contributing to an immunosuppressive microenvironment. Therefore, targeting lactate secretion could lead to a better GBM prognosis by preventing the PMT (Wang et al., 2023). Moreover, the inhibition of GLUT1 expression prevents lactate secretion, acting on glycolysis pathway, and leading to a reduction in immunosuppressive TAMs (Li et al., 2024). Additionally, lactate induces epigenetic reprogramming of immune cells through histone lactylation, altering gene expression to support tumor growth (Zhang et al., 2019). Particularly, Wang et al. demonstrated that such an epigenetic modification is correlated to CD47 upregulation and the phagocytosis alteration in GBM cells. These data suggest that the epigenetic reprogramming related to lactate secretion could be an effective target to increase the effectiveness of immuno-therapies against GBM (Wang et al., 2024).

GBM shows alterations also in glutamine metabolism, contributing to its increased tumor growth (Fu et al., 2024). Indeed, glutamine deprivation is correlated to reduced cell growth rate, while high levels are characteristic of GBM mesenchymal profile, contributing to treatment resistance (Ekici et al., 2022).

Recently, it has been demonstrated that epidermal growth factor receptor (EGFR), through the upregulation of glutamate dehydrogenase 1 (GDH1), supports glutamine metabolism in GBM, promoting cell proliferation (Yang et al., 2020).

Acetate is an astrocyte-specific bioenergetic substrate, exploited as an alternative source of energy in the brain. Recently, it has been demonstrated that acetate is also used by GBM as energetic metabolite; indeed, GBM cells show an increased acetate production, also leading to reactive astrogliosis in the surrounding microenvironment (Kim et al., 2024). GBM cells retain the glial capacity to oxidize acetate; however, they are able to co-oxidate both acetate and glucose to sustain a higher proliferative rate (Mashimo et al., 2014). The upregulation of acyl-CoA synthetase short chain family member 2 (ACSS2), which converts acetate to acetyl-CoA, is typical in GBM and it is correlated to a worst prognosis in GBM patients (Comerford et al., 2014; Mashimo et al., 2014).

It has been shown that GBM subtypes are characterized by different metabolic features, associated with a specific clinical outcome. For instance, from a metabolic perspective, mesenchymal GBM tumors show typical susceptibilities that are not observed in the other GBM subtypes (Wang et al., 2021). Through a proteogenomic study, Wang et al. characterized different types of GBMs from brain samples, dividing them into three clusters: proneural-like, mesenchymal-like and classical-like. This study reported that the proneural-like cluster is characterized by a higher abundance of acetylated proteins involved in OXPHOS, while the mesenchymal-like is enriched for glycolysis (Wang et al., 2021).

Metabolic differences between mesenchymal and proneural subtypes have been found also in glioma stem cells (GSCs) (Marziali et al., 2016). Interestingly, it has been demonstrated that GSCs share similarities with the respective GBM cells. Marziali et al. showed that GSCs with a proneural-like phenotype are characterized by high levels of N-acetylaspartate and glutamine, both of which are associated with neural metabolism and mitochondrial function, suggesting an oxidative phenotype. On the contrary, GSCs with a mesenchymal-like phenotype show a downregulation of N-acetylaspartate and glutamine, suggesting a shift towards glycolytic metabolism, which is often associated with aggressive and rapidly proliferating cells (Marziali et al., 2016). In addition, other studies revealed that mesenchymal subtype shows a higher glycolytic activity as compared to proneural one, with an increased expression of aldehyde dehydrogenase 1 family member A3 (ALDH1A3), a key enzyme associated with different metabolic processes, including glucose metabolism (Figure 1) (Mao et al., 2013; Xu et al., 2024).

In conclusion, GBM subtypes exhibit distinct metabolic profiles that reflect their aggressiveness. Mesenchymal subtype shows higher glycolytic activity and a more aggressive phenotype, while proneural subtype relies more on OXPHOS, correlating with a less aggressive behaviour and a better clinical outcome (Figure 1).

### 2.2 Lipid metabolism

Lipids are structural components of cell membranes, nevertheless they are also involved in cellular signalling and energy reserve. Due to their rapid proliferation, cancer cells modulate their lipid metabolism adapting to increased energy request (Shakya et al., 2021).

**FIGURE 2:**
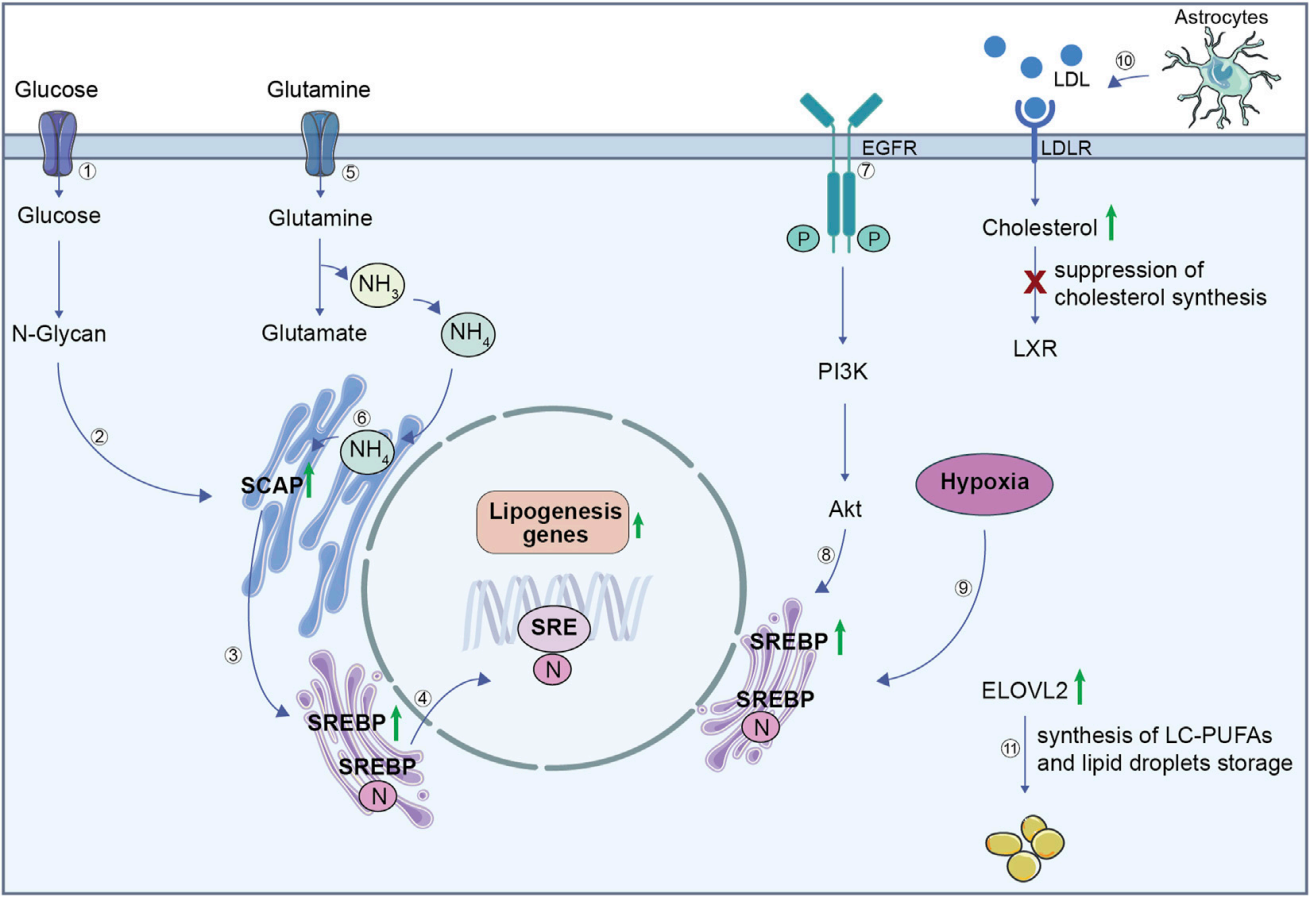
Lipid metabolism in GBM cells. (1) Glucose uptake contributes to the synthesis of N-glycan (2), which stabilizes SCAP. (3) SCAP facilitates the activation of SREBP, which translocates to the nucleus (4) to stimulate the expression of lipogenesis genes. (5) Glutamine is imported inside the cell and its metabolism produces glutamate and ammonium (NH₄⁺). (6) Ammonium binds to SCAP transmembrane domains, triggering a conformation change. Consequently, SCAP induces translocation of SREBP from the endoplasmic reticulum to the Golgi where it releases its active N-terminal fragment. The N-terminal domain goes into the nucleus, binds to the SRE to activate lipogenesis genes transcription. This regulation integrates signals from both glucose and glutamine metabolism to ensure efficient lipogenesis, which is critical for GBM growth and survival. (7) EGFR activation initiates the PI3K/Akt signalling pathway, (8) which reinforces SREBP activity and drives lipogenesis. (9) GBM hypoxic environment upregulates SREBP, leading to an increase in lipogenesis genes. (10) LDL uptake via LDLR increases cholesterol levels but suppresses cholesterol synthesis through LXR inactivation. (11) ELOVL2 upregulation in GBM promotes the synthesis of long-chain polyunsaturated fatty acids and supports lipid droplet storage. SCAP, SREBP cleavage-activating protein; SREBP, sterol regulatory element-binding protein; SRE, sterol regulatory element; EGFR, epidermal growth factor receptor; PI3K/Akt, phosphoinositide 3-kinase/protein kinase B; LDL, low-density lipoprotein; LDLR, LDL receptor; LXR, liver X receptor; ELOVL2, elongation of very long chain fatty acids protein 2.

Guo et al. demonstrated that GBM enhances fatty acid (FA) synthesis to foster tumor growth and proliferation (Guo et al., 2009a). Increased glucose and glutamine consumption promotes lipid production through sterol regulatory element-binding protein (SREBP)/SREBP-cleavage activating protein (SCAP) pathway (Figure 2) (Kou et al., 2022).

In addition, SREBP-1 leads to an increased glutamine consumption and lipogenesis through the upregulation of ASCT2 expression, a glutamine transporter. In turn, releasing ammonia, glutamine stimulates SREBP-1 activation, establishing a loop that improves glutamine metabolism and lipid biosynthesis (Zhong et al., 2024).

Recent studies revealed that the oncogenic pathway epidermal growth factor receptor (EGFR)/Phosphatidylinositol 3-Kinase (PI3K)/Protein kinase B (AKT) activates SREBP-1, that is directly involved in FA synthesis; indeed, SREBP-1 inactivation, through a pharmacological inhibitor or shRNA, reduces GBM proliferation *in vitro* and *in vivo* (Guo et al., 2009b; Geng et al., 2016). Interestingly, SREBP shows a key role in FA and cholesterol metabolism also under hypoxia, thus its inhibition impairs lipid biosynthesis in hypoxic cancer cells (Figure 2). Moreover, Lewis et al. demonstrated that stearoyl-CoA desaturase (SCD) and fatty acid-binding protein 7 (FABP7), involved in mono-unsaturated FA (MUFA) biosynthesis and FA uptake and trafficking, respectively, are correlated with SREBP function (Lewis et al., 2015). SCD1 is also crucial for GSCs lipid metabolism, and MUFAs synthesis inhibition impairs their stem cell features, reducing tumor growth in a GBM mouse model (Pinkham et al., 2019). Furthermore, mitogen-activated protein kinase (MEK)/extracellular signal-regulated kinase (ERK) signalling pathway is involved in GSCs vulnerability; indeed, MEK/ERK inhibits AMP-activated protein kinase (AMPK) activity that, through a modification in lipid composition, shields GSCs from lipotoxicity (Eyme et al., 2023).

Compared to healthy brain tissues, GBM stores more FA as an energy reservoir and to foster the *de novo* lipid synthesis supporting cell proliferation (Taib et al., 2019; Shakya et al., 2021). It has been shown that elongation of very long chain fatty acids protein 2 (ELOVL2), which catalyses the first reaction of long-chain FA elongation cycle, is upregulated in GSCs and it promotes their survival (Gimple et al., 2019). This supports the hypothesis that tumor growth is strengthen by the production of long-chain polyunsaturated acids (LC-PUFAs) (Figure 2).

The central nervous system (CNS), with about the 20% of total body cholesterol, is considered the most cholesterol-rich organ. Interestingly, GBM cells reduce their own cholesterol synthesis, while they exploit astrocyte-derived cholesterol to support their proliferation (Deshmukh et al., 2021). The strong responsiveness that GBM exhibits towards liver X receptor (LXR) agonists, can be explained by its reliance on cholesterol uptake (Figure 2). In particular, LXR regulates cholesterol homeostasis in the CNS and, its BBB-permeable agonist LXR-623 promotes cell death and blocks tumor growth in a GBM mouse model (Courtney and Landreth, 2016; Villa et al., 2016).

Interestingly, through metabolomic and lipidomic analyses, Wang et al. demonstrated that lipid composition is deeply different among GBM subtypes. Tumors with a mesenchymal subtype show a high level of triacylglycerols and decreased phosphatidylcholines, while proneural subtypes show a large amount of very long chain fatty acid lipids (VLCFAs) and LC-PUFAs. Moreover, proneural tumors exhibit an overexpression of Acyl-CoA synthetase long chain family member 6 (ACSL6) and of docosahexaenoic acid (DHA) as compared to mesenchymal tumors (Wang et al., 2021).

Notably, a GBM metabolic reprogramming involving the *de novo* synthesis of fatty acids promotes PMT. In particular, malic enzyme 2 (ME2), which catalyzes pyruvate formation from malic acid, upregulates mesenchymal markers, downregulating proneural genes. ME2 reprograms lipogenesis through AMPK/SREBP-1/ACSS2 signaling, by reducing mitochondrial ROS production and AMPK phosphorylation, leading to SREBP-1 maturation and ACSS2 lipogenesis pathway promotion (Yang et al., 2021; Wu et al., 2024).

Therefore, alterations in the *de novo* lipid synthesis seem to be a potential susceptibility target for GBM; however further studies are required to better understand the weak knot in lipid metabolic reprogramming that could be an effective therapeutic target.

### 2.3 Nucleotide metabolism

**FIGURE 3:**
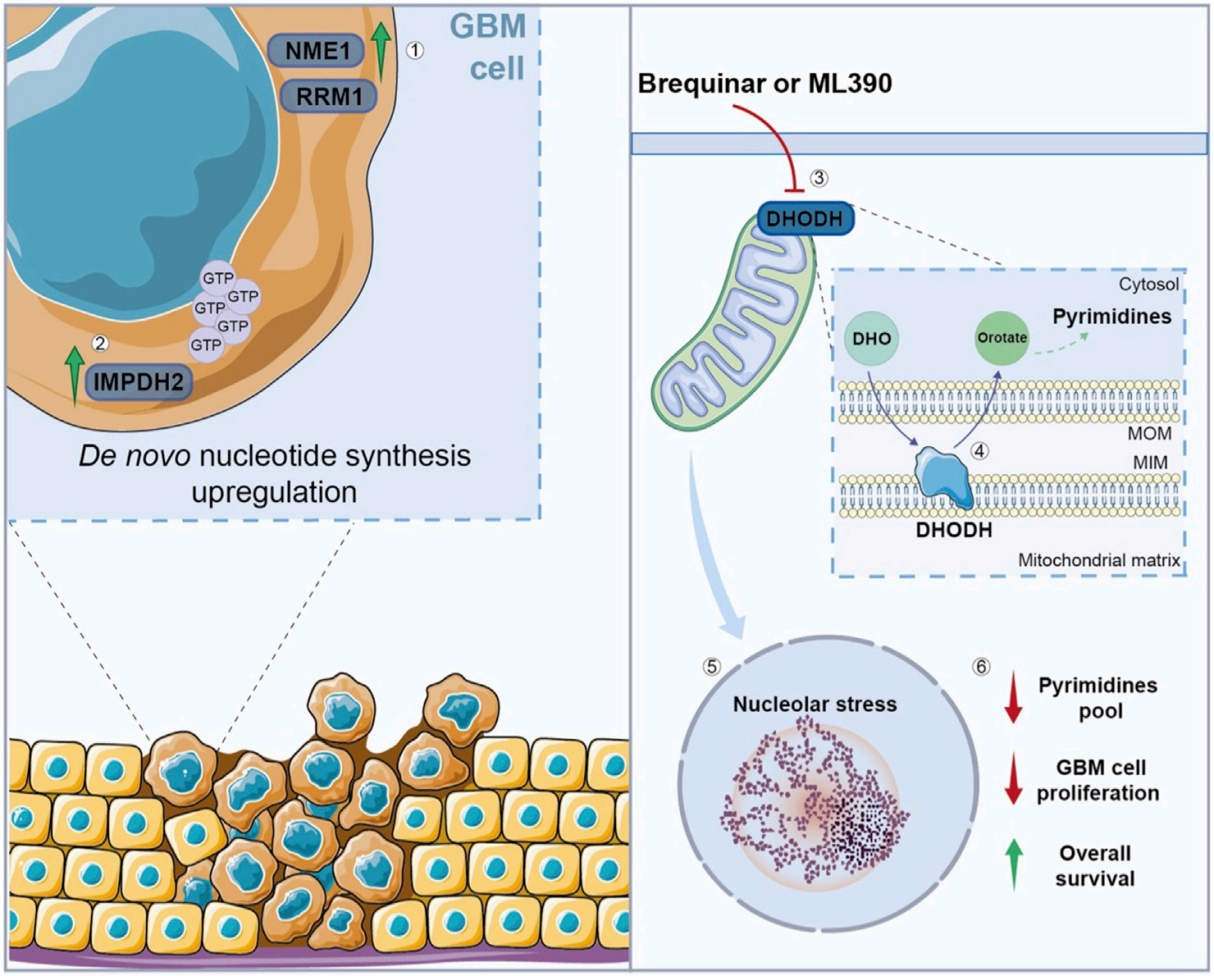
Nucleotide metabolism in GBM cells. (1) GBM cells show an upregulation of enzymes involved in nucleotide synthesis, such as NME1, RRM1, and (2) IMPDH2, which is involved in GTP synthesis, promoting *de novo* nucleotide synthesis. (3) The inhibition of DHODH, through Brequinar or ML390, (4) leads to alterations in the *de novo* pyrimidine biosynthesis, (5) resulting in nucleolar stress, and subsequent reduction in pyrimidines pool and GBM cell proliferation, while (6) the overall survival increases after the treatment. NME1, nucleoside diphosphate kinase 1; RRM1, ribonucleotide reductase catalytic subunit M1; IMPDH2, inosine monophosphate dehydrogenase-2; GTP, guanosine triphosphate; DHODH, dihydroorotate dehydrogenase; DHO, Dihydroorotate; MOM, mitochondrial outer membrane; MIM, mitochondrial inner membrane.

Nucleotides are the principal structural components of nucleic acids, but they are also implicated in energy metabolism and cellular signalling. Their synthesis occurs through two main pathways: *de novo* nucleotide synthesis and nucleotide salvage (Zhou and Wahl, 2019). The *de novo* synthesis of purines and pyrimidines is extremely energy-consuming and requires amino acids and ribose; instead, the nucleotide salvage pathway needs less energy and uses purines and pyrimidines derived from nucleotide catabolism (Bernhard et al., 2023). Differentiated cells use the salvage pathway to produce nucleotides, while proliferative cells typically rely on *de novo* synthesis due to their increased purines and pyrimidines request. Thus, GBM cells reshape their nucleotide metabolism in order to increase progression, upregulating the *de novo* nucleotide synthesis to sustain their proliferation (Ghannad-Zadeh and Das, 2021). Using a large-scale targeted proteomics platform, Nakamizo et al. confirmed that the *de novo* pyrimidine synthesis is one of the most enriched pathways in GBM patients. Indeed, ribonucleotide reductase catalytic subunit M1 (RRM1) and nucleoside diphosphate kinase 1 (NME1), encoding for enzymes involved in nucleotide synthesis, are upregulated in GBM (Figure 3) (Nakamizo et al., 2022). Furthermore, GBM upregulates inosine monophosphate dehydrogenase-2 (IMPDH2), a rate-limiting enzyme involved in guanosine triphosphate (GTP) synthesis, improving RNA polymerase I and III transcription (Figure 3) (Kofuji and Sasaki, 2020).

Recently, Spina et al. demonstrated that the inhibition of dihydroorotate dehydrogenase (DHODH), involved in the *de novo* pyrimidine biosynthesis, reduces tumor growth, prolonging the overall survival in a GBM orthotopic mouse model (Spina et al., 2022). Notably, DHODH is required for ribosomal DNA (rDNA) transcription and its inhibition causes nucleolar stress in GBM cells, reducing their proliferation (Figure 3) (Lafita-Navarro et al., 2020).

Wang et al. demonstrated that the inhibition of purine synthesis, by altering the intracellular purine pool, reduces GSCs proliferation and tumor progression *in vivo*. However, the growth of differentiated GBM cells is not significantly influenced by the modulation of the enzymes involved in purine biosynthesis. The transcription factor MYC is implicated in GSCs *de novo* purine synthesis pathway and modulated by PI3K–AKT pathway, regulating the expression of enzymes involved in purine biosynthesis. Accordingly, GBM patients with higher expression of *de novo* nucleotide synthesis enzymes show a poor prognosis and dismal clinical outcome (Wang et al., 2017). Moreover, GBM could reprogram nucleotide salvage pathway to reuse purines and pyrimidines bases, as demonstrated by increased expression of related enzymes (Cortes Ballen et al., 2024). Metabolic reprogramming in GBM also affects nucleotide catabolic pathways, since the expression levels of enzymes involved in these pathways are dysregulated (Cortes Ballen et al., 2024).

In conclusion, GBM can reprogram nucleotide metabolism to increase its proliferation and progression. Therefore, the modulation of nucleotide metabolic pathways could be a therapeutic target or an adjuvant treatment for GBM patients. However, the *de novo* biosynthesis has been more studied and explored as compared to the salvage pathway, and few insights are available on purines and pyrimidines catabolism, hence new studies addressing these features are needed to gain a clearer understanding of nucleotide metabolism in GBM.

### 2.4 Iron metabolism

Iron serves as vital element for maintaining physiological processes, including DNA synthesis, cell proliferation, oxidative stress and energy production (Manz et al., 2016; Gao et al., 2019). Due to its multifaceted function, iron represents a pivotal element in the metabolic reprogramming of GBM, sustaining tumor aggressiveness and proliferation. Indeed, GBM cells exhibit an increased demand for iron to meet their metabolic needs (Schonberg et al., 2015). It has been observed that GSCs require a higher amount of iron as compared to non-stem cells due to their capacity of self-renewal. Knocking down ferritin, the protein responsible for storing iron in GSCs, impairs their proliferation capacity, indicating that iron is an essential element for their survival and growth (Schonberg et al., 2015).

**FIGURE 4:**
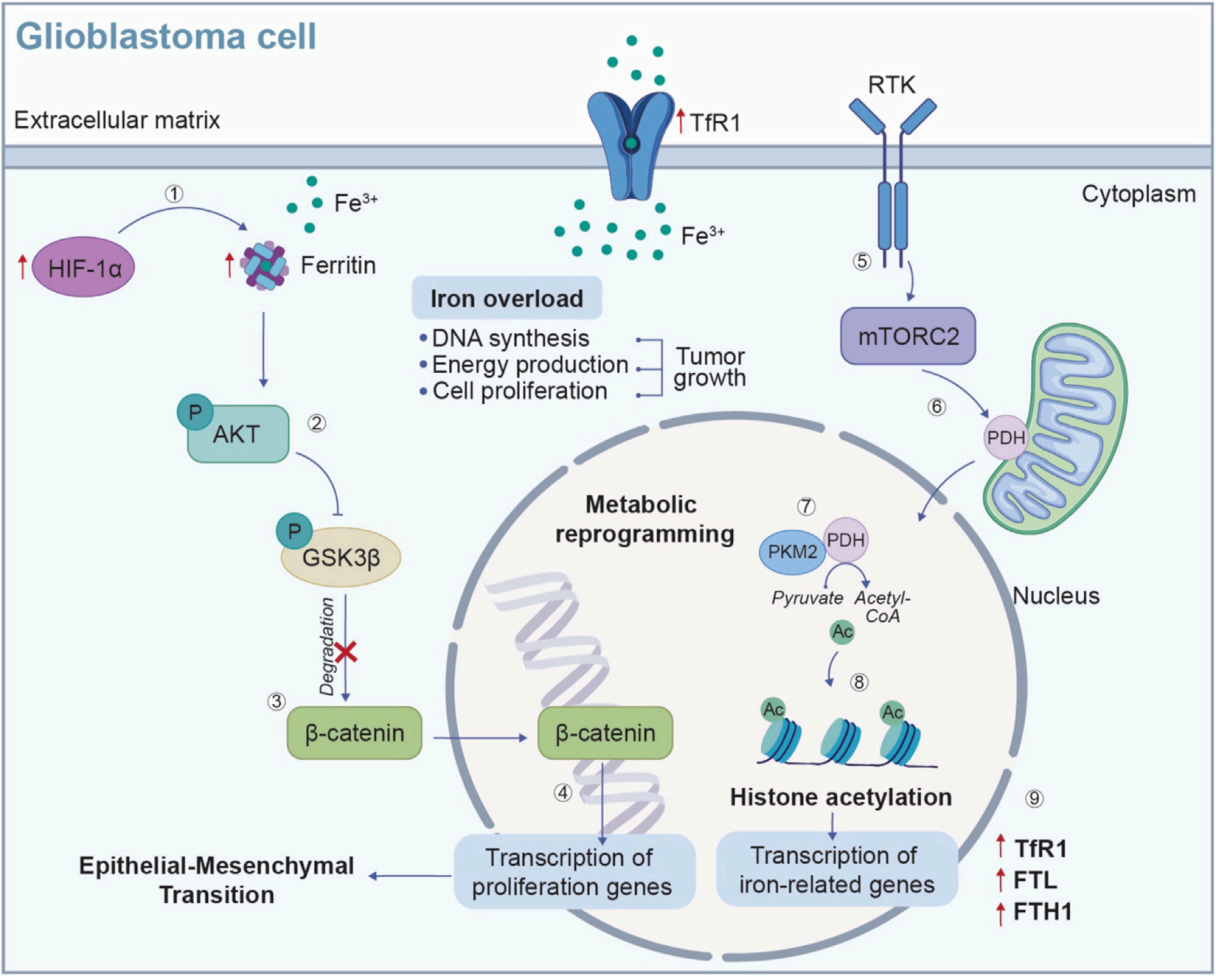
Iron metabolic reprogramming in GBM cells. Iron is an essential contributor for metabolic reprogramming in cancer cells, supporting key processes such as DNA synthesis, energy production and cell proliferation. (1) In the hypoxic TME, the expression of HIF-1α is increased, leading to an upregulation of ferritin (FTL/FTH) and TfR1, which enhances iron uptake and storage to sustain GBM cell growth. (2) The accumulation of iron triggers the activation of AKT signaling pathway, which phosphorylates and inhibits GSK3β. (3) This inhibition prevents the degradation of β-catenin, (4) allowing β-catenin to accumulate and to translocate into the nucleus, where it binds to transcription factors and activates genes involved in cell proliferation. Additionally, the RTK/mTORC2 signaling pathway contributes to the metabolic reprogramming of GBM cells. (5-6) Activation of mTORC2 by the RTK promotes PDH activity in the mitochondria where, (7) by interacting with the transcription co-activator PKM2 enhances the conversion of pyruvate into acetyl-CoA. (8) In the nucleus, acetyl-CoA serves to histone acetylation, (9) a process that promotes the transcription of iron-related genes, such as TfR1, FTL and FTH1, further increasing iron uptake and driving tumor growth. TME: tumor microenvironment; HIF-1α: hypoxia-inducible factor 1 subunit alpha; FTL, ferritin light chain; FTH, ferritin heavy chain; TfR1, transferrin receptor 1; AKT, protein kinase B; GSK3β, Glycogen synthase kinase-3 beta; RTK, receptor tyrosine kinase; mTORC2, mTOR2 complex; PDH, pyruvate dehydrogenase; PKM2, pyruvate kinase isozymes M2.

It is reasonable to hypothesize that GBM cells modulate the expression of proteins involved in iron uptake to sustain tumor growth. Indeed, it has been shown that the expression of transferrin receptor 1 (TfR1), also known as cluster of differentiation 71 (CD71), is increased in GBM cells (Figure 4) (Rosager et al., 2017). As a matter of fact, TfR1 and ferritin, the main players involved in iron metabolism, are increased in correlation to the tumor grade, meaning that their increase is associated with poorer clinical outcomes (Liu et al., 2020). Interestingly, the differential expression of proteins involved in iron uptake between different GBM subtypes reflects the distinct prognoses associated with these phenotypes. Vo et al. discovered a commensal symbiosis between proneural and mesenchymal GSCs (Vo et al., 2022). In this scenario, proneural GSCs sustain mesenchymal GSCs releasing dopamine and transferrin, thus promoting the growth of neighbouring cells, enhancing iron uptake and increasing their proliferation capacity. Additionally, released dopamine increases TfR1 expression in mesenchymal GSCs, further promoting intracellular iron content (Vo et al., 2022).

Typical hallmarks of the TME, such as hypoxia and acidic environment, can influence iron metabolism, leading to an enhanced uptake and storage of iron, thereby facilitating uncontrolled cell proliferation and tumor growth (Wang et al., 2016). GBM cells also upregulate the levels of ferritin, the primary storage form of iron in the CNS, to increase iron sequestration (Liu et al., 2020). Li and collaborators observed increased levels of ferritin light chain (FTL) in TAMs within GBM microenvironment. The upregulation of FTL suppress calcium-independent phospholipase A2β (iPLA2β), whose activity, under physiological condition, prevents cells damage by inhibiting lipid peroxide accumulation (Li et al., 2023). This suppression promotes the polarization of TAMs towards the M2-like phenotype, which contributes to an immunosuppressive TME, thereby facilitating tumor progression and immune evasion (Li et al., 2023). Ferritin upregulation is associated with the epithelial-mesenchymal transition (EMT) and chemoresistance in glioma under hypoxic conditions, by activating the AKT/Glycogen synthase kinase-3 beta (GSK3β)/β-catenin signalling pathway (Figure 4) (Liu et al., 2020).

The maintenance of homeostatic iron levels is guaranteed by the iron regulatory protein 1 (IRP1, also known as ACO1), which regulates the expression of ferritin and transferrin at the post-transcription levels (Katsarou and Pantopoulos, 2020).

An additional piece of evidence, highlighting the involvement of iron metabolism in GBM metabolic reprogramming, analyses the role of epigenetic regulators that influence tumor growth. In an orthotopic GBM mouse model, mTOR2 complex (mTORC2) activation has been shown to induce H3 histone acetylation, promoting the transcription of iron-related genes, including TfR1, FTL and ferritin heavy chain (FTH1), which in turn enhances the survival of GBM cells (Figure 4) (Masui et al., 2019).

Iron also plays a significant role in maintaining the redox balance within GBM cells, participating in Fenton reaction in which ferrous iron (Fe^2+^) reacts with hydrogen peroxide (H_2_O_2_) to generate hydroxyl radicals. This means that excessive amount of free iron could lead to uncontrolled reactive oxygen species (ROS) production, the main responsible for DNA damage, contributing to genomic instability, an important hallmark of cancer (Torti and Torti, 2013). Moreover, these radicals could attack polyunsaturated fatty acids (PUFAs) in cell membranes, initiating lipid peroxidation, suggesting that the interplay between iron and lipid metabolism is another important aspect of GBM metabolic reprogramming.

The complex interplay between iron levels, metabolic demand and TME conditions underscores the importance of developing targeted therapies aimed at disrupting iron metabolism in GBM, by understanding the mechanism through which GBM cells manipulate iron levels.

## 3 Metabolic reprogramming in glioblastoma as a mechanism of therapy resistance

Metabolic reprogramming is one of the main strategies that GBM employs to evade the antitumoral effects of standard therapies. GBM can modulate its metabolism in order to reduce cellular stress produced by chemotherapy, allowing GBM cells survival and proliferation in spite of the treatment; thus, there has been a growing interest in the study of metabolic reprogramming for its potential impact in therapy resistance (Hsu, 2023).

**FIGURE 5:**
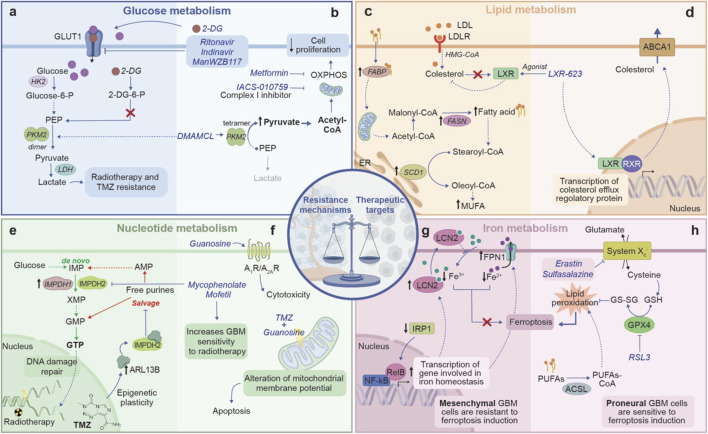
The double-edged sword of metabolic reprogramming in GBM, showing the balance between resistance mechanisms and therapeutic vulnerability. **(A)** *Glucose metabolism in therapy resistance:* GBM cells increase glucose uptake through GLUT1 and funnel it towards glycolysis and lactate production, leading to resistance to radiotherapy and chemotherapy. **(B)** *Glucose metabolism as therapeutic target:* pharmacological agents such as 2-DG and inhibitor of GLUT1 transporter can alter glucose metabolism, reducing cancer cell proliferation. By promoting PKM2 tetramerization, DMAMCL shifts cellular metabolism towards OXPHOS. Drugs such as metformin and IACS-010759 disrupt OXPHOS, thereby limiting cell proliferation. **(C)** *Lipid metabolism in therapy resistance:* upregulation of FABP increases the shuttle of fatty acids to various cellular compartments, including mitochondria, where fatty acids are converted in MUFAs by SCD1, providing an accessible energy source that promotes cell surviving. Moreover, GBM cells rely on exogenous cholesterol for survival and suppress LXR ligand synthesis, which enables to access a nearly limitless supply of cholesterol to fuel their growth. **(D)** *Lipid metabolism as therapeutic target:* LXR agonist promotes LXR activity which through a negative feedback regulation, decreases cholesterol levels by promoting the expression of efflux cholesterol transporters ABCA1. **(E)** *Nucleotide metabolism in therapy resistance:* through the upregulation of enzymes like IMPDH1 and IMPDH2, GBM increases the *de novo* synthesis of GTP, essential for DNA damage repair. Moreover, TMZ administration induces epigenetic modifications that upregulate the expression of ARL13B, which binding to IMPH2, suppress the purine salvage pathway. The shift towards the *de novo* synthesis gives GBM cells a self-sufficient means of producing nucleotide, supporting both their growth and their abilities to evade therapies. **(F)** *Nucleotide metabolism as therapeutic target:* targeting IMPDH2 with Mycophenolate Mofetil disrupts GTP synthesis, sensitizing GBM to radiotherapy. Additionally, the combination of TMZ with guanosine destabilizes mitochondrial membrane potential, inducing apoptosis, while the activation of adenosine receptors further amplifies cytotoxicity. **(G)** *Iron metabolism in therapy resistance:* GBM cells exhibit decreased expression of IRP1, leading to the upregulation of genes involved in iron homeostasis such as FPN1 and LCN2. This upregulation promotes iron export from the cell, reducing the intracellular iron pool and consequently ferroptosis. **(H)** *Iron metabolism as therapeutic target:* inhibition of System Xc^−^, which supports GSH synthesis, or inhibition of GPX4 using RSL3 disrupts GBM antioxidant defenses. This disruption promotes lipid peroxidation and triggers ferroptosis. ABCA1, ATP-binding cassette sub-family A member 1; ARL13B, ADP-rybosylation factor-like protein 13B; 2-DG, 2-deoxyglucose; FABP, fatty acid binding protein; FPN1, ferroportin 1; GSH, glutathione; GPX4, Glutathione peroxidase 4; IMPDH, inosine monophosphate dehydrogenase; LXR, liver X receptor; LCN2, lipocalin 2; MUFA, monounsatured fatty acids; PKM2, pyruvate kinase M2; SCD1, stearoyl-CoA Desaturase 1; TMZ, temozolomide. Dashed arrows indicate indirect interactions. Solid arrows indicate direct interactions.

Notably, glycolytic GBM cells show greater resistance to radiation; indeed, it has been demonstrated that downregulation of the glycolytic enzyme Hexokinase 2 (HK2) increases GBM sensitivity to radiotherapy (Figure 5A) (Vartanian et al., 2016; Zhou and Wahl, 2019). Interestingly, lactate dehydrogenase (LDHA) and anaerobic glycolysis contribute to GBM resistance, not only to radiotherapy, but also to chemotherapy. As a matter of fact, it has been demonstrated that glycolysis inhibition in GBM cell lines, through LDHA gene silencing, increases their radiation sensitivity and their response to TMZ (Koukourakis et al., 2017). As evidence that the Warburg effect is highly involved in chemotherapy resistance, Velpula et al. showed that the inhibition of pyruvate dehydrogenase kinase (PDK) and the stimulation of mitochondrial enzyme pyruvate dehydrogenase (PDH) are able to revert the Warburg effect, leading to GBM cytotoxicity and to an increased sensitivity to TMZ (Velpula et al., 2017).

By analysing the characteristics of a GSCs subpopulation involved in resistance and recurrencies, Zhou et al. demonstrated that these cells increase glycolysis flux and showed reduced mitochondrial respiration, thus better tolerating hypoxia. These cells preferentially localize in hypoxia niches and are typically resistant to TMZ (Zhou et al., 2011). Reshaping their metabolism may represent a potential strategy against residual GBM cells.

Recently, new studies also explored the role of lipid metabolism rewiring in GBM therapy resistance, revealing a crucial impact on GBM progression. It has been demonstrated that TMZ-resistant GBM cells show increased long-chain fatty acids levels and reduced PUFAs as a result of upregulation of fatty acid synthase (FASN), and a downregulation of delta-6-desaturase (D6D), the key enzyme for PUFA biosynthesis (Figure 5C) (Kao et al., 2023). The increased FA levels in TMZ-resistant GBM have been also correlated to an improved uptake and transport, which is correlated to the enhanced expression of FA transport proteins and of FABP (Figure 5C) (Caragher et al., 2020; Tanase et al., 2022). Moreover, Dai et al. demonstrated that TMZ-resistant GBM cells exhibit increased SCD1 levels, while its downregulation resensitizes GBM cells, revealing SCD1 as a potential target to increase the chemotherapy efficacy in GBM patients (Figure 5C) (Dai et al., 2017).

A recent study revealed that purine metabolism is involved in radiotherapy resistance; surely, resistant GBM exploit purine metabolites to support DNA double-stranded breaks (DSBs) repair caused by radiations. Moreover, high expression of inosine monophosphate dehydrogenase 1 (IMPDH1), an enzyme implicated in the *de novo* GTP synthesis, is correlated with a shorter survival time in GBM patients. GTP synthesis inhibition increases GBM cells sensitivity to radiotherapy and improves radiation effects in GBM orthotopic models (Figure 5E) (Zhou et al., 2020). The *de novo* purine biosynthesis is also implicated in GBM chemoresistance. Indeed, ADP-ribosylation factor-like protein 13B (ARL13B), which regulates the adaptation to TMZ, interacts with IMPDH2, a key enzyme for purine biosynthesis. Thus, the inhibition of ARL13B-IMPDH2 interaction or the block of IMPDH2 activity through mycophenolate mofetil, increase GBM sensitivity to TMZ (Figure 5E) (Shireman et al., 2021).

Regarding the role of iron metabolism in treatment resistance, Tong et al. demonstrated that the overexpression of transferrin receptor 2 (Tfr2), involved in iron uptake, enhances TMZ efficacy, through an increased production of ROS (Tong et al., 2023). Lan Y. and colleagues identified IRP1 as key factor for GBM patient prognosis. They observed that in TMZ-resistant GBM cells, IRP1 expression is significantly reduced, while the levels of lipocalin 2 (LCN2) and ferroportin (FPN1), two important iron-transported proteins that regulate intracellular iron levels, are increased (Figure 5G) (Lan et al., 2023). The study identified nuclear factor kappa B subunit 2 (NFKB2), a component of the noncanonical nuclear factor-κB (NF-kB) pathway, as a critical downstream target of IRP1, while the knockdown of NFKB2 in IRP1-deficient cells reverses TMZ resistance (Lan et al., 2023). It has been reported that GBM cells also upregulate cystine transporter solute carrier family 7 member 11 (SLC7A11), protecting cells from oxidative stress and reducing chemosensitivity (Hu et al., 2020). Indeed, SLC7A11 knockdown decreases GSH expression, enhancing TMZ effects in GBM cells (Polewski et al., 2016).

Therefore, accumulating evidence suggests that metabolic reprogramming has a key role in GBM therapy resistance. Further studies are needed to investigate these mechanisms and to better understand how revert them in order to increase GBM sensitivity to treatment.

## 4 Metabolic reprogramming in glioblastoma as a new therapeutic target

Recently, many studies focused on targeting GBM metabolism as a potential treatment strategy (Zhao et al., 2024). Glucose uptake inhibition has been investigated as a new therapeutic approach, altering GBM metabolism. It has been proven that WP1234, a compound that releases 2-Deoxy-d-glucose (2-DG), a competitive analogue of glucose, when metabolized, suppresses GBM cells viability (Pajak et al., 2022). Dimethylaminomicheliolide (DMAMCL) reduces GBM cell proliferation, rewiring aerobic glycolysis through the activation of pyruvate kinase 2 (PKM2) (Figure 5B) (Guo et al., 2019). Moreover, acting on glucose uptake and transporters represents a promising approach against GBM growth. GLUT inhibitors ritonavir and indinivar, reduce glucose intake and GBM cell viability, while ManWZB117, another GLUT1 inhibitor, is effective in hampering GSCs proliferation, but not in inhibiting tumor progression (Figure 5B) (Shibuya et al., 2015; Azzalin et al., 2017; Garcia et al., 2021).

Interestingly, also mitochondria could be a therapeutic target for GBM. Metformin, a widely used anti-diabetic drug, induces GBM cell death and reduces tumor growth, inhibiting OXPHOS and decreasing ATP production (Figure 5B) (Sesen et al., 2015). Clinical trials for Metformin in combination with chemotherapy or radiotherapy are in phase II for GBM treatment (Bi et al., 2020). It has been demonstrated that also phenformin, a Metformin analogue, shows a potential as tumor treatment, indeed it decreases GSCs markers and, in combination with TMZ, reduces tumor growth (Jiang et al., 2016). Furthermore, IACS-010759, an inhibitor for complex I of the mitochondrial electron transport chain (ETC), significantly reduces GBM proliferation, limiting GBM cell energy and inhibiting nucleotide synthesis (Figure 5B) (Molina et al., 2018). Notably, blocking Drp1, a cytosolic GTPase involved in mitochondrial fission, through Mdivi-1, strongly reduces the proliferation of GBM cells. Given that Drp1 is strongly expressed in GSCs, the inhibition of mitochondrial fission could be effective in GSCs targeted therapy (Table 1) (Xie et al., 2015; Wu et al., 2022).

**TABLE 1:** Effects of metabolic drugs on GBM models and the related clinical trials.

Metabolic reprogramming	Metabolic drug	Effects on GBM models	References	Clinical trials
*Glucose metabolism*	*WP1234*	Inhibits glycolysis and reduces GBM cell viability	Pajak et al. (2022)	—
	*DMAMCL*	Rewires aerobic glycolysis, suppressing GBM cells proliferation and colony formation	Guo et al. (2019)	In phase I, in China and Australia, for GBM treatment Trial ID: actrn12616000228482
	*Ritonavir and Indinavir*	Decrease glucose consumption and lactate production, inhibiting GBM cells growth	Azzalin et al. (2017)	—
	*ManWZB117*	Blocks GLUT1 and inhibits self-renewal capacity in GSCs	Shibuya et al. (2015)	—
	*Metformin*	Decreases mitochondrial-dependent ATP production and oxygen consumption, reducing GBM cells proliferation and delaying tumor growth in a xenograft GBM mouse model	Sesen et al. (2015)	In phase II, in South Korea and Canada, for GBM treatment, in combination with TMZ and radiotherapy Trial ID: NCT02780024 and NCT03243851
	*Phenformin*	Inhibits mitochondrial complex I, decreases the expression of stemness and mesenchymal markers in GSCs, inhibiting tumor growth and prolonging overall survival of an orthotopic GBM mouse model	Jiang et al. (2016)	—
	*IACS-010759*	Blocks complex I of the mitochondrial electron transport chain, inhibits proliferation and induces apoptosis in models of brain cancer, reducing tumor growth *in vivo*	Molina et al. (2018)	—
	*Mdivi-1*	Inhibits Drp1 and mitochondrial fission, reducing GSCs proliferation	Xie et al. (2015)	—
*Lipid metabolism*	*LXR-623*	Inhibits cholesterol uptake inducing cell death in patient-derived GBM cells and causing tumor regression in GBM mouse models	Villa et al. (2016)	—
	*Archazolid B*	Alters cholesterol homeostasis, causing excessive accumulation of free cholesterol and leading to cytotoxicity effects in GBM cells	Hamm et al. (2014)	—
*Nucleotide metabolism*	*Guanosine*	Promotes cytotoxicity and induces apoptosis in TMZ-treated GBM cells	Oliveira et al. (2017)	—
*Iron metabolism*	*Sulfasalazine*	Inhibits system Xc- and increases TMZ cytotoxicity in GBM cells	Ignarro et al. (2016)	In phase I, in Norway, for recurrent GBM treatment Trial ID: NCT04205357
	*Erastin*	Sensitizes GBM cells to TMZ, by inducing ferroptosis	Chen et al. (2015)	—
	*FAC*	Through iron overload, reduces metabolic turnover and induces cytotoxic effects in proneural GBM cells	D’Aprile et al. (2024)	—

Since alterations in lipid metabolism are typical features of GBM progression, new studies on drugs targeting lipid metabolites have been conducted. As a matter of fact, LXR-623 inhibits cholesterol uptake, while archazolid B blocks cholesterol recycling, thus they could be effective targets to reduce GBM invasiveness (Figure 5D; Table 1) (Garcia et al., 2021). Meanwhile, considering nucleotide metabolism, it has been demonstrated that guanosine treatment in combination with TMZ leads to apoptosis in GBM cells via adenosinergic system, suggesting purines as a potential adjuvant treatment in association with chemotherapy (Figure 5F; Table 1) (Oliveira et al., 2017).

Furthermore, CNS holds a high amount of PUFAs and a low activity for antioxidant enzymes, as such GBM is susceptible to lipid peroxidation and oxidative stress (Rao et al., 2000; Lu et al., 2022). These features sustain the hypothesis that ferroptosis could be a successful strategy for GBM treatment. Ferroptosis is an iron-dependent cell death, correlated to ROS production and the subsequent lipid peroxidation (Dixon et al., 2012). Several mediators involved in iron metabolism have been studied as ferroptosis inducers. Notably, such a type of cell death can be triggered by acting on system Xc- or on GPX4 though small molecules (i.e., erastin, sulfasalazine, RSL3), otherwise by modulating lipid metabolism balance or increasing intracellular iron levels with ferric ammonium citrate (FAC, Figure 5H) (Liang et al., 2019). Recently, it has been demonstrated that ferroptosis induction reduces GBM tumor growth, and, both erastin and sulfasalazine intensify GBM cells sensitivity to TMZ (Figure 5H) (Chen et al., 2015; Ignarro et al., 2016; Mitre et al., 2022). However, the effects of ferroptosis induction could be limited by the hypoxic TME and by the GBM heterogeneity (Kapralov et al., 2020; Zhuo et al., 2022). GBM subtypes differently tolerate ferroptosis induction; indeed, GBM cells with a proneural profile reduce their metabolic turnover and viability after FAC or erastin administration, while cells with a mesenchymal profile do not show significant variations (Figures 5G, H). Such a different response can be related to a better and more effective antioxidant system in mesenchymal tumors, supporting the resistance to ferroptosis inducers (D'Aprile et al., 2024). However, ferroptosis inducers, such as RSL3 and erastin, have been used to sensitize mesenchymal GSCs to ferroptotic stress (Table 1) (Vo et al., 2022). Such a discrepancy could be explained taking into account that GSCs require high levels of iron to sustain their proliferation rate, as discussed above.

Besides GBM heterogeneity, a limitation of metabolic therapy is the off-target toxic effects on healthy cells, as they partially share metabolic pathways with tumor cells. Moreover, another challenge associated with metabolic drugs is the adaptability of GBM; indeed, the tumor may use other nutrients or modify its metabolism to overcome the toxic effects of therapies, leading once again to drug resistance mechanisms. Thus, to mitigate tumor metabolic plasticity it could be crucial to monitor the tumor and its dynamic metabolic changes, adapting metabolic therapy as needed (Xiao et al., 2023).

Therefore, targeting metabolism could be a promising approach for GBM treatment, especially in combination with radiotherapy and chemotherapy. However, it is necessary to consider the heterogeneity of GBM by analysing the metabolic differences between proneural, mesenchymal, classical tumors to achieve personalized therapy, and to develop strategies limiting the side effects of metabolic drugs.

## 5 Conclusion

GBM exhibits the ability to reprogram metabolism to support proliferation and invasiveness. The complex GBM TME also affects tumor metabolism, promoting GBM adaptability in inhospitable conditions. This deadly cancer is able to act on specific metabolic pathways to reverse the effects triggered by therapy, inducing resistance. However, GBM subtypes show different ability to rewire metabolism and to remodel physiological processes. Typically, mesenchymal tumors show metabolic alterations that are not detectable in classical and proneural subtypes, leading to increased aggressiveness and resistance. Thus, studies focusing on GBM metabolic mechanisms should consider the intrinsic variability between subtypes.

Notably, metabolic reprogramming is a double-edge sword for GBM, indeed, on the one hand is a strength to increase therapeutic resistance, on the other hand metabolism could be also a weakness for this tumor. GBM can alter specific metabolic pathways to enhance its proliferation and aggressiveness; however, reverting these modifications could effectively neutralize these advantages, inhibiting tumor growth through drugs targeting GBM metabolism.

Many studies revealed that drugs affecting glucose, lipid or nucleotide metabolism show positive effects in reducing tumor proliferation and therapy resistance. Ferroptosis could be also effective in reducing GBM growth and in improving therapy efficacy, although GBM cells could implement specific mechanisms to tolerate the effects caused by the induction of iron-dependent cell death.

Targeting metabolism could be an effective strategy to counteract GBM growth, but further investigation into the pathways involved in metabolic reprogramming across GBM subtypes is crucial to identify specific therapeutic tools.
